# Dynamic Modelling of Tooth Deformation Using Occlusal Kinematics and Finite Element Analysis

**DOI:** 10.1371/journal.pone.0152663

**Published:** 2016-03-31

**Authors:** Stefano Benazzi, Huynh Nhu Nguyen, Ottmar Kullmer, Kornelius Kupczik

**Affiliations:** 1 Department of Cultural Heritage, University of Bologna, Ravenna, Italy; 2 Department of Human Evolution, Max Planck Institute for Evolutionary Anthropology, Leipzig, Germany; 3 Department of Biomaterials, Max-Planck-Institute of Colloids and Interfaces, Potsdam, Germany; 4 Department of Palaeoanthropology and Messel Research, Senckenberg Research Institute, Frankfurt am Main, Germany; 5 Max Planck Weizmann Center for Integrative Archaeology and Anthropology, Max Planck Institute for Evolutionary Anthropology, Leipzig, Germany; Second University of Naples, ITALY

## Abstract

**Background:**

Dental biomechanics based on finite element (FE) analysis is attracting enormous interest in dentistry, biology, anthropology and palaeontology. Nonetheless, several shortcomings in FE modeling exist, mainly due to unrealistic loading conditions. In this contribution we used kinematics information recorded in a virtual environment derived from occlusal contact detection between high resolution models of an upper and lower human first molar pair (M^1^ and M_1_, respectively) to run a non-linear dynamic FE crash colliding test.

**Methodology:**

MicroCT image data of a modern human skull were segmented to reconstruct digital models of the antagonistic right M^1^ and M_1_ and the dental supporting structures. We used the Occlusal Fingerprint Analyser software to reconstruct the individual occlusal pathway trajectory during the power stroke of the chewing cycle, which was applied in a FE simulation to guide the M_1_ 3D-path for the crash colliding test.

**Results:**

FE analysis results showed that the stress pattern changes considerably during the power stroke, demonstrating that knowledge about chewing kinematics in conjunction with a morphologically detailed FE model is crucial for understanding tooth form and function under physiological conditions.

**Conclusions/Significance:**

Results from such advanced dynamic approaches will be applicable to evaluate and avoid mechanical failure in prosthodontics/endodontic treatments, and to test material behavior for modern tooth restoration in dentistry. This approach will also allow us to improve our knowledge in chewing-related biomechanics for functional diagnosis and therapy, and it will help paleoanthropologists to illuminate dental adaptive processes and morphological modifications in human evolution.

## Introduction

Dental biomechanics is a pivotal research area in dentistry to test materials for tooth restoration and to analyse the causes of dental failures [1, 2]. In biology and palaeontology, dental biomechanics is the basis for understanding the relationship between tooth morphology, functional demands and constraints, and evolutionary adaptive processes in the masticatory apparatus of living and extinct animals [3–7]. One of the virtual and numerical tools of dental biomechanics is finite element (FE) analysis which is commonplace in dental industry [8, 9] and has increasingly been used in evolutionary biological studies [7, 10–14].

Nonetheless, it has been recently observed that several shortcomings in FE modeling exist [15], one of which are unrealistic loading conditions. Occlusal loads are usually simplified as point loads applied parallel or oblique with respect to the long axis of the tooth [9, 16–18], but do not take into account the actual topography of the occlusal surface.

Benazzi and colleagues [15] pointed out that during antagonistic occlusal contact wear facets should be considered and loaded in different directions and to different extents. The authors used the Occlusal Fingerprint Analyser (OFA) software [19] to determine the individual contact areas during the power stroke, i.e. the phase of the chewing cycle when upper and lower dentitions meet and antagonistic crowns usually develop wear facets due to attritional and abrasive loss of enamel.

The OFA software is developed to virtually test, record and verify the real physiological directions of the individual power stroke movements, also reflected in the patterns and spatial positions of physical wear facets on the tooth crowns [19]. After loading surface models of antagonistic crowns aligned in maximum intercuspation into the OFA software, the user determines an assumed pathway of the power stroke with beginning and end points for each direction of power stroke movements [19]. The software then moves the models of the lower jaw along the determined pathway and as soon as tooth-to-tooth contact occurs the models are automatically guided along their relief surfaces towards the next defined path-point of the power stroke, generating a real relief guided trajectory. The accuracy of the individual kinematics can be assessed afterwards by comparing the original contact patterns (wear facet position) on the crowns with the results of virtual contact areas, detected during the calculation of the pathway trajectory. The individuals`trajectory is recorded per user-defined time step. Based on OFA results, Benazzi and colleagues [15] selected for their FE analysis the power stroke contact areas (collision surfaces) recorded in three power stroke-representative time steps, i.e., during the incursive phase I, at maximum intercuspation contact (centric occlusion), and in the excursive phase II to evaluate the stress distribution in teeth during occlusion.

Even though this approach is by far more realistic than simply reducing the occlusal force to point loads, it explains just three static moments of a sequentially changing stress scenario in a chewing cycle.

Recent contributions tried to overcome this issue by combining FE analysis and information on the human chewing process acquired either by using a jaw motion-capture system from live patients [20], or by simulating digitally the closing phase of the masticatory cycle. The latter was done by moving vertically upwards and medially the lower molar toward the upper molar to achieve maximum intercuspation [21].

Despite the obvious merits (e.g., more realistic simulations), these approaches are based on patient teeth, which impose several crucial limitations: 1) often only the external surface of the crown and not the entire tooth morphology is considered (e.g. [20]); 2) the low resolution of the underlying image data to build a volumetric mesh or coarse resolution of the volumetric mesh itself (e.g. [21]); 3) the boundary conditions, which are applied on the tooth itself and thus can lead to unrealistic local stresses (e.g. [21]). Alternatively, the complete cranium or mandible is considered [22–24], but again to the detriment of volumetric mesh resolution and dental structure accuracy. In general, most contributions developed so far, which combine FE analysis and dynamic loading conditions, do not employ high resolution models to accurately identify the changes in occlusal contact areas during the simulation. Yet, these are needed to assess the force distribution on the tooth surface with cascading effects on stress/strain distributions in the whole tooth and dental supporting tissues.

In this contribution we used the full individual kinematic pathway reconstructed and recorded in a virtual environment during the power stroke (i.e., the trajectory of the mandibular molar relative to its antagonist) to run a non-linear dynamic FE crash colliding test in a high resolution digital model of the upper and lower first molars and their dental supporting tissues. To our knowledge this approach provides the most realistic simulation of how chewing forces are distributed over the tooth surface, which is fundamental for an accurate evaluation of the stresses and strains occurring in teeth and dental supporting tissues under physiological loading conditions.

## Materials and Methods

### Micro-Computed Tomography Scanning, Segmentation and 3D Reconstruction

In 2011, the skull of a young adult female (ID = S23) selected from the archaeological skeletal sample collected by Rudolf Poech in South Africa in 1907–1909 [25], currently stored at the Department of Anthropology of the University of Vienna, was micro-CT scanned at the Vienna Micro-CT Lab, Department of Anthropology, University of Vienna, with a Viscom X8060 μCT (scan parameters: 130kV, 100 mA). Volume data were reconstructed using isometric voxels of 60μm.

**Fig 1:**
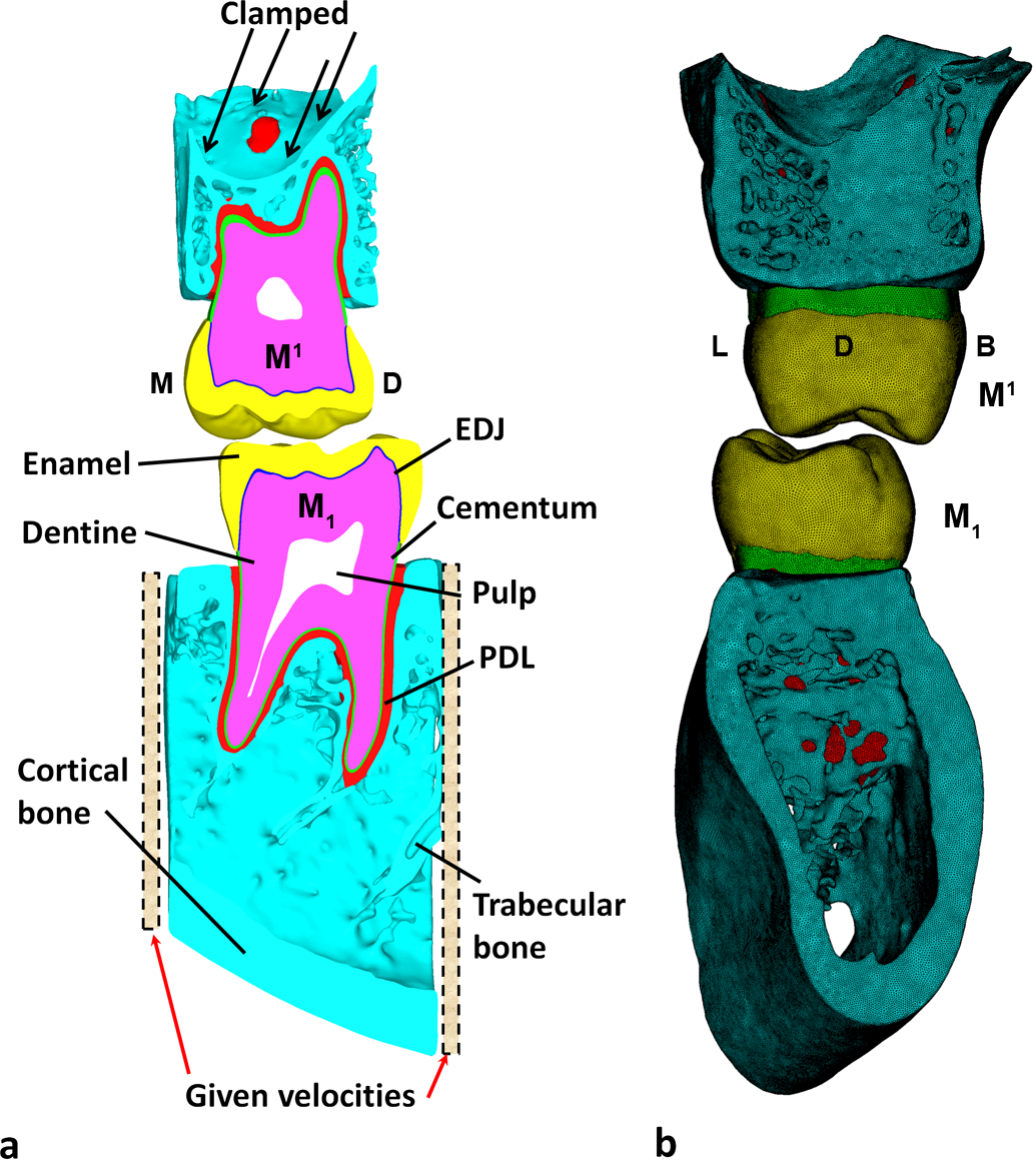
Digital models of the upper and lower first molars. a) Segmented model of lower right first molar (M_1_, bottom) and upper right first molar (M^1^, top). Section is through the mesio-distal axis of the teeth. b) FE mesh in distal view. PDL = periodontal ligament; EDJ = Enamel-dentine junction. B = buccal; D = distal; L = lingual; M = mesial.

The micro-CT image data was cropped mesially and distally to the socket of the right mandibular and maxillary first molar (RM_1_ and RM^1^, respectively) to reduce the size of the digital models. Segmentation of the dental and bone tissues (enamel, dentine, pulp, trabecular and cortical bone) was carried out in Avizo 7 (Visualization Sciences Group Inc.). The cementum was modeled as a 0.135 mm and 0.195 mm thick layer that envelops the external root surface of the lower and upper molars, respectively (average cementum thickness from [26] (Fig 1). The periodontal ligament (PDL) was created by filling the gap between the cementum and the alveolar socket, thus varying in thickness (Fig 1). Moreover, a layer of 0.1 mm was created between enamel and dentine [27] to reflect the cushioning zone between the two tissues (enamel-dentine junction, hereafter called EDJ), following recent findings that highlight the importance of this layer to accommodate mechanical stresses [14, 28, 29]. The final refinement of the digital model (i.e., triangles optimization) was carried out in Geomagic Studio 2012 (Geomagic, Inc) (Fig 1A).

**Fig 2:**
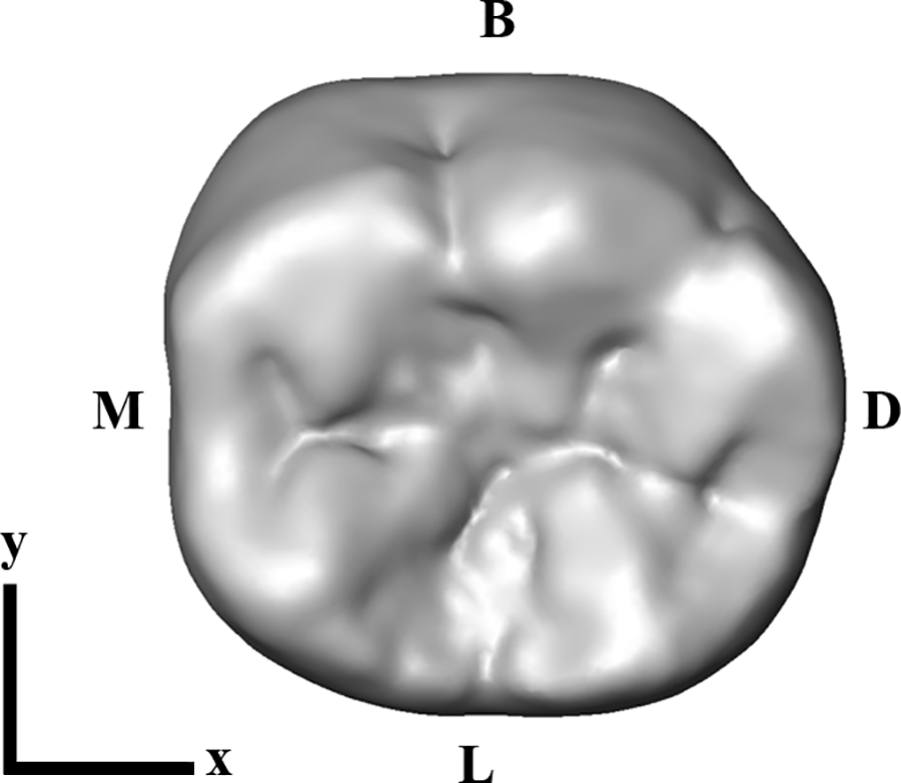
Occlusal view of the oriented lower right first molar (RM_1_). The tooth is aligned with the cervical plane parallel to the xy-plane of the Cartesian coordinate system, and the mesial side parallel to the y-axis. B = buccal; D = distal; L = lingual; M = mesial.

In order to define an orientation system for the digital models, the best-fit plane through the cervical outline at the enamel-cementum junction (i.e., cervical plane) of the RM_1_ was computed in Geomagic Studio 2012. The RM_1_ was aligned with the cervical plane parallel to the xy-plane of the Cartesian coordinate system and rotated around the z-axis, so that the mesial side was parallel to the y-axis (Fig 2) [15].

### Occlusal Kinematics

**Fig 3:**
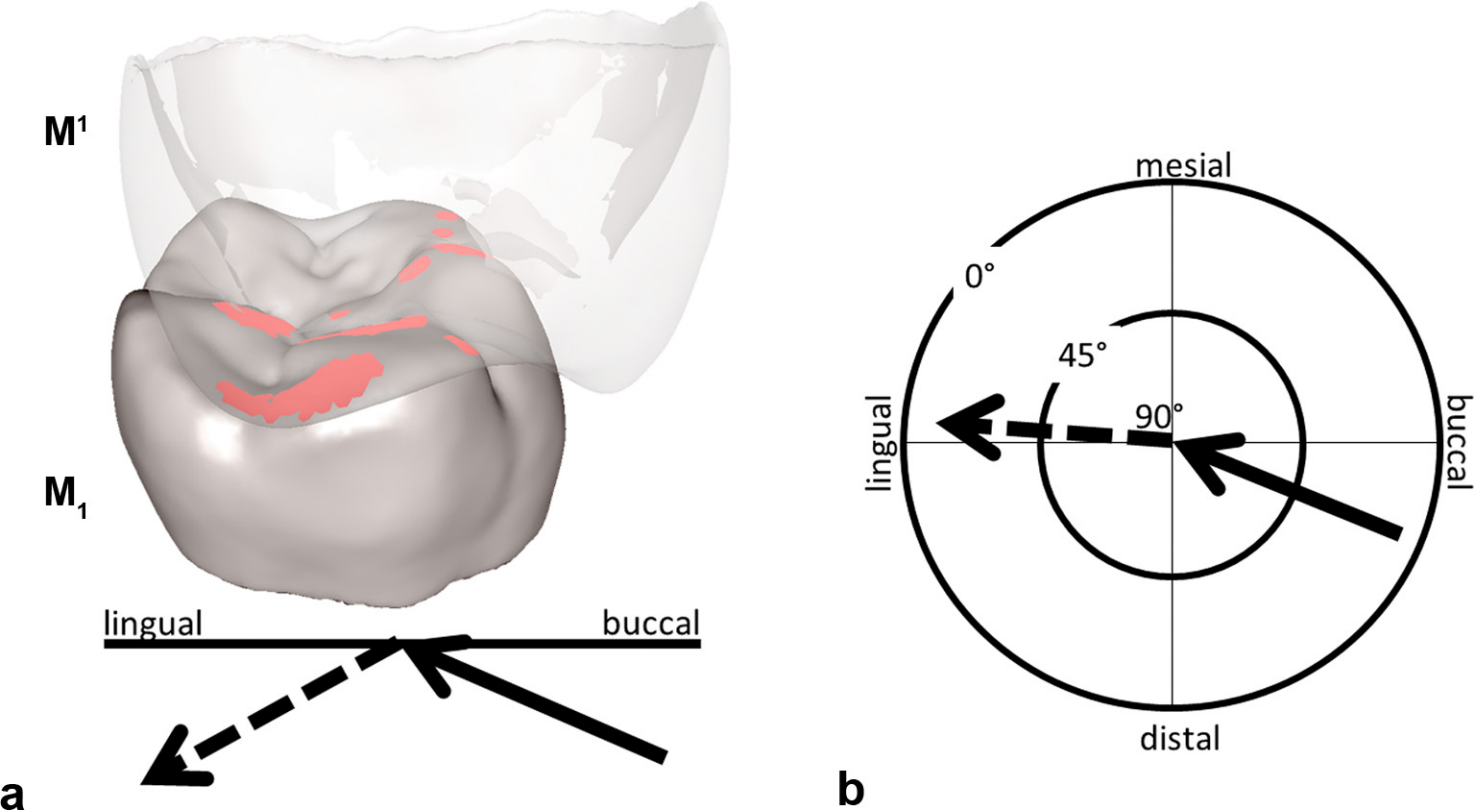
Collision detection for RM_1_ with the antagonist RM^1^ in the Occlusal Fingerprint Analyser software (OFA) during maximum intercuspation contact. a) The RM^1^ is transparent to show the collision (red areas) in the occlusal surface of the RM_1_; the recorded power stroke pathway trajectory of the RM_1_ (summarized by the two arrows) is subdivided into an incursive (black arrow = phase I) and an excursive (dashed arrow = phase II) vector. b) The mastication compass visualizes the spatial orientation of phase I and II. The length of the two arrows informs about the inclination angle (after [31]).

The dental surface models of the RM_1_/RM^1^ were imported into the OFA software and the RM^1^ was adjusted in maximum intercuspation position on the oriented RM_1_. Following general knowledge about functional anatomy and biomechanics of mastication [30], an assumed pathway for the power stroke was set for the lower molar coming from distobuccally upward and slightly anteriorly until maximum intercuspation, and then downward towards slightly mesiolingually. Thanks to collision detection, deflection and break free algorithms implemented in the OFA software, one model is allowed to move against the other, ultimately providing a kinematic analysis of the surface contacts between RM_1_ and RM^1^ during the power stroke [15]. In detail, after having set a starting point and an endpoint for the movements, as soon as the lower and upper teeth collide, the travelling model (RM_1_) is deflected and guided by the antagonistic crown relief producing its individual power stroke trajectory. Contact areas are marked in different colors and the occlusal pathway trajectory is recorded, informing on the occlusal movements during the incursive (phase I) and excursive (phase II) of the power stroke (Fig 3A and 3B; S1 Video). The calculated collisions were compared to the wear facets (macrowear pattern) present on the occlusal surface of the real tooth in order to verify the accuracy of the generated trajectory. The starting and endpoints of the trajectory calculation were repeated three times until the resulting collisions matched with the functional macrowear pattern on the original specimen. The verified individuals`pathway trajectory was exported as a list of Cartesian coordinates (x,y,z) and used in the FE software to guide the RM_1_ in the crash colliding test.

**Fig 4:**
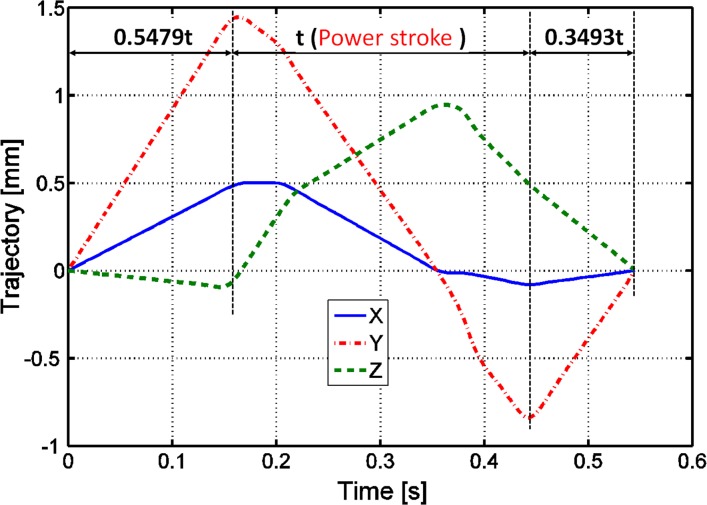
The trajectory of the power stroke. Trajectory (x-, y- and z-axes) obtained for the RM_1_ during the chewing cycle (i.e., close + power stroke + open) in the OFA software scaled to the duration of the power stroke (t = 289 ms).

The OFA software allows for recording the occlusal pathway trajectory (or power stroke, i.e. phase I and phase II), but the duration of the chewing cycle (i.e., closing phase + power stroke + opening phase) is automatically, yet unphysiologically determined by the software [19]. Therefore, we used information gathered from the literature to calibrate the duration of each of the phases of the chewing cycle. Specifically, modern human jaw movements recorded with a magnet-sensing jaw-tracking system suggest that the power stroke may last about 289 ms (measured from [30]; his Fig 1–15). Since in the OFA simulation the closing and opening phases were computed to be 0.5479 and 0.3493 times the power stroke, respectively (Fig 4), we estimated a total movement time of 548.311ms.

### Volumetric Mesh Generation and FE Analysis

The surface models were imported into HyperWorks Software (Altair Engineering, Inc.), where volumetric meshes were created (Fig 1B). Two simulations with 4-noded 2,846,131 tetrahedral elements (hereafter called “coarse” mesh) and 8,832,739 tetrahedral elements (hereafter called “fine” mesh), respectively, were carried out to verify the convergence of the results. For the hard tissues (enamel, dentine, EDJ, cementum, bone), tetrahedral elements with 6 rotational and translational degrees of freedom (DOF) at each node were used [32], hence improving accuracy of the simulation. For the soft tissues (pulp, PDL) each node had 3 DOFs and tetrahedral elements with constant pressure formulation for incompressible material were used. The number of elements arranged across the 0.15–0.85 mm PDL thickness varies between 2 and 8, accordingly. Only a single layer of elements was arranged across the 0.1 mm thick EDJ. Adaptive meshes were not required to capture the thin geometric features of PDL and EDJ, because in both cases the mesh size satisfied the mesh quality requirements (e.g., warpage, aspect ratio).

Material property values (elastic modulus *E*, Poisson’s ratio and density ν) were collected from the literature (Tables 1 and 2). The same mechanical properties and density were used for cortical and trabecular bone (hereafter called “bone”), following the suggestion by Hall [43].

**Table 1:** Elastic properties of isotropic materials.

Material	*E* (GPa) ^c^	Poisson's ratio	References
**Enamel**	84.1	0.3	[33]
**Dentine**	24.5	0.31	[34]
**EDJ**^a^	51.35	0.3	Average between enamel and dentine
**Pulp**	0.002	0.45	[35]
**Cementum**	15.5	0.31	[34]
**PDL**^b^	0.0689	0.45	[36]
**Bone (cortical and trabecular)**	13.7	0.3	[37]

^a^Enamel-dentine junction

^b^Periodontal ligament

^c^Elastic modulus

**Table 2:** Density of tissues (ρ) and speed of sound (Vsound).

	Average density ρ (kg/m^3^)	Reference	Vsound* [mm/s]
**Bone (cortical and trabecular)**	1990	[38]	3.04E+06
**Dentine**	2140	[38]	3.98E+06
**Cementum**	2063	[39]	3.23E+06
**Enamel**	2958	[39]	6.19E+06
**EDJ**	2840	[40]	4.93E+06
**PDL**	1100	[41]	4.87E+05
**Pulp**	1000	[41]	8.71E+04

*The material speed of sound is calculated based on indication provided by [42]; see Eq (1)

For the EDJ, *E* and ν were computed as the average values between enamel and dentine [14]. All materials were considered homogeneous, linearly elastic and isotropic, which is an evident simplification but it is comparable with current studies in dental biomechanics [44].

Regarding the boundary constraints, the superior surface of the maxilla was totally fixed in X, Y and Z directions, as during mastication the maxillary bone does not move. In contrast, since the mandible moves during mastication and is subjected to torsional loading and sagittal bending [45, 46], for all the surface nodes of the mesial and distal surfaces of the mandible section the velocities calculated in X, Y and Z directions were imposed simultaneously (see below for details about the velocity).

The explicit scheme was used to solve the governing equations [47]. This method is efficient for highly non-linear problems, especially when dealing with complex contacts and large deformations. Moreover, it is particularly reasonable for FE models with several million elements, for which the implicit scheme has problems with computational cost, convergence or contact failure [48].

In FE non-linear simulations, the movement time (see above) is subdivided in multiple time steps (∆t), which should be small to guarantee 1) a linearly approximated behavior of the structure and 2) a stability of algorithm. In general, ∆t is automatically calculated by the software. The initial default value is ∆t = 2.1E-09s, but to save computational time ∆t was scaled to 5.0E-08s for the open and close phases (mass scaling, i.e. the software increases the density of the components of the model; see [49]). The default ∆t assigned by HyperWorks was used for the power stroke.

While the time scaling (i.e., increasing velocity) does not affect the linear elastic material used for the FE models (i.e., there is no strain rate dependency), high scaled velocity may introduce nonrealistic dynamic effects that can alter the accuracy of the solution. However, limiting scaled velocity to less than 1% of the wave speed in the simulated materials does not affect significantly the accuracy [49]. After several trials with different scaling factors (145, 100, 80, 60 and 50), the time scaling factor was set to Fsc = 50, because it assured the smallest acceptable kinetic energy in the closing phase. The maximum velocity after scaling was still smaller than 1% speed of sound (Vsound) of pulp tissue (Table 2), the latter was computed following [42]:

$$\text{Vsound}=\sqrt{\left[\frac{\text{E}\left(1-\text{v}\right)}{\rho \left(1+\text{v}\right)\left(1-2\text{v}\right)}\right]}$$ (1)

where E = Young’s modulus (MPa), v = Poisson’s ratio and ρ = density (kg/mm^3^).

Moreover, we introduced a diagonal damping matrix **C**, proportional to mass matrix **M**, in the dynamic equation to damp spurious oscillations:

$$Mü\left(t\right)+C\dot{u}\left(t\right)+\text{K}u\left(t\right)\;=\;F\left(t\right)$$ (2)

$$C=\left(\frac{2B}{T}\right)\;M$$ (3)

where: **K** is the stiffness matrix; the time-dependent vectors **u**(*t*), $\dot{u}$(*t*) and **ü**(*t*) are nodal displacements, velocities and accelerations, respectively; **F**(*t*) is time-dependent external load vector; *B* is the relaxation value (default value 1); *T* is the period to be damped (imposing *T* = 0.0035s, close to the highest frequency of the model from PDL).

The direct integration method used in the HyperWorks solver package is derived from Newmark time integration scheme. Velocity and displacement at time *t*_*n*+1_ = *t*_*n*_ + Δt are calculated:

$${\dot{u}}_{n+1}\;={\dot{u}}_{n}+\left(1-\gamma \right)\Delta \text{t}{\ddot{u}}_{n}+\gamma \Delta \text{t}{\ddot{u}}_{n+1}$$ (4)

$$u_{n+1}\;=\;u_{n}+\Delta \text{t}{\dot{u}}_{n}+\left(0.5-\beta \right){\Delta \text{t}}^{2}{\ddot{u}}_{n}+\beta {\Delta \text{t}}^{2}{\ddot{u}}_{n+1}$$ (5)

The integration scheme used by HyperWorks’ solver RADIOSS is based on the central difference integration algorithm with coefficients β = 0 and γ = 0.5. It is conditionally stable when the time step Δt is small enough to integrate accurately the response in the highest frequency component.

A penalty type formulation [32], which is accounted for by the type 7 interface of HyperWorks software, allows sliding between a master surface (occlusal surface of the M^1^ enamel) and a set of slave nodes (occlusal surface of the M_1_ enamel). This interface has spring stiffness when a slave node penetrates the interface gap. At a contacted node the elastic contact force has two components: normal and tangent. The normal component of the contact force is a function of contact-related parameters as:

$$F_{n}=K\;\left(\frac{gp}{g-p}\right)$$ (6)

with

- g is the gap value (i.e., to determine when contact between slave nodes and master surface occurs), which by default was defined by the software HyperWorks as one tenth of the smallest side of all master element segments (g = 6.636E-03 mm);

- p is the penetration of a slave node into a master segment (g ≥ *p* ≥ 0). Penetration is checked in each time step of the power stroke. When a penetration is registered, the force proportional to the penetration depth (6) is applied to resist the penetration;

- *K* is the interface spring stiffness: *K* = *min*(*K*_*m*_, *K*_*S*_, *K*_*min*_).

The master stiffness is computed as $K_{m}\;=\;St_{FAC}\times Bu\times \frac{S^{2}}{V}$;

The slave stiffness is an equivalent nodal stiffness computed as $K_{S}\;=\;St_{FAC}\times B\times \sqrt[{3}]{V}$;

Minimum stiffness is *K*_*min*_ = 84100, equal to Young’s modulus of enamel.

In the above formulae *St*_*FAC*_ = 1 (stiffness factor); *Bu* is the bulk modulus of enamel material; *S* is the master element area; *V* is the master element volume. Overall, the interface spring stiffness is related to material parameters, ultimately favoring the efficacy of the penalty method, because the stiffness of contacting enamels has the same order of magnitude.

We introduced a Coulomb friction law, using a friction coefficient μ = 0.2, a value found for wet conditions [50]. The tangent component of the contact force is then calculated as:

$$F_{t}=\mu F_{n}$$ (7)

A nodal contact force is the resultant force of a normal component (6) and a tangent component (7). Summation of contact forces at each node produced by the enamel contact interface provides the contact force between the lower and upper enamel caps, which is equivalent to the chewing force. During the power stroke values of the chewing force are changing according to the changes of contact area size and spatial position between enamel caps. Therefore, the power stroke chewing force is time dependent, whereas in both the closing and opening phase the chewing force is equal to zero.

Gravity force was constantly applied on all parts of the model, even though we observed that gravity effect is not important for this simulation.

Results for were qualitatively and quantitatively evaluated according to the first maximum principal stresses criterion for brittle materials (as stress wave propagation is fundamental to investigate dynamic effects of chewing load on materials) [51, 52], but for the PDL (i.e., soft tissue) the first principal strain was evaluated.

### Sensitivity of FE Methodology

When experimental data are not available, fundamental checks should be undertaken to reduce the error of the explicit simulation (S1–S5 Figs). First, the mesh size of the FE model must be fine (namely small) to prevent unphysical stress even when the velocity is high. To address this issue, the average mesh size value is h (height) = 0.13mm, with a total number of 8,832,739 tetrahedral elements. Second, the quality of the simulation’s results was estimated through the evaluation of the 1) energy balance (S1 Fig); 2) ratio of kinetic energy to internal energy (S2 Fig); and 3) time history of kinetic energy, internal energy and contact energy [48] (S1 Text). Moreover, differences between the two simulations (using the “coarse” and “fine” meshes, respectively) were <5% for displacement (S3 Fig) and internal energy (S4 Fig), confirming the convergence of the results. Only results obtained using the “fine” mesh are reported below.

## Results

**Fig 5:**
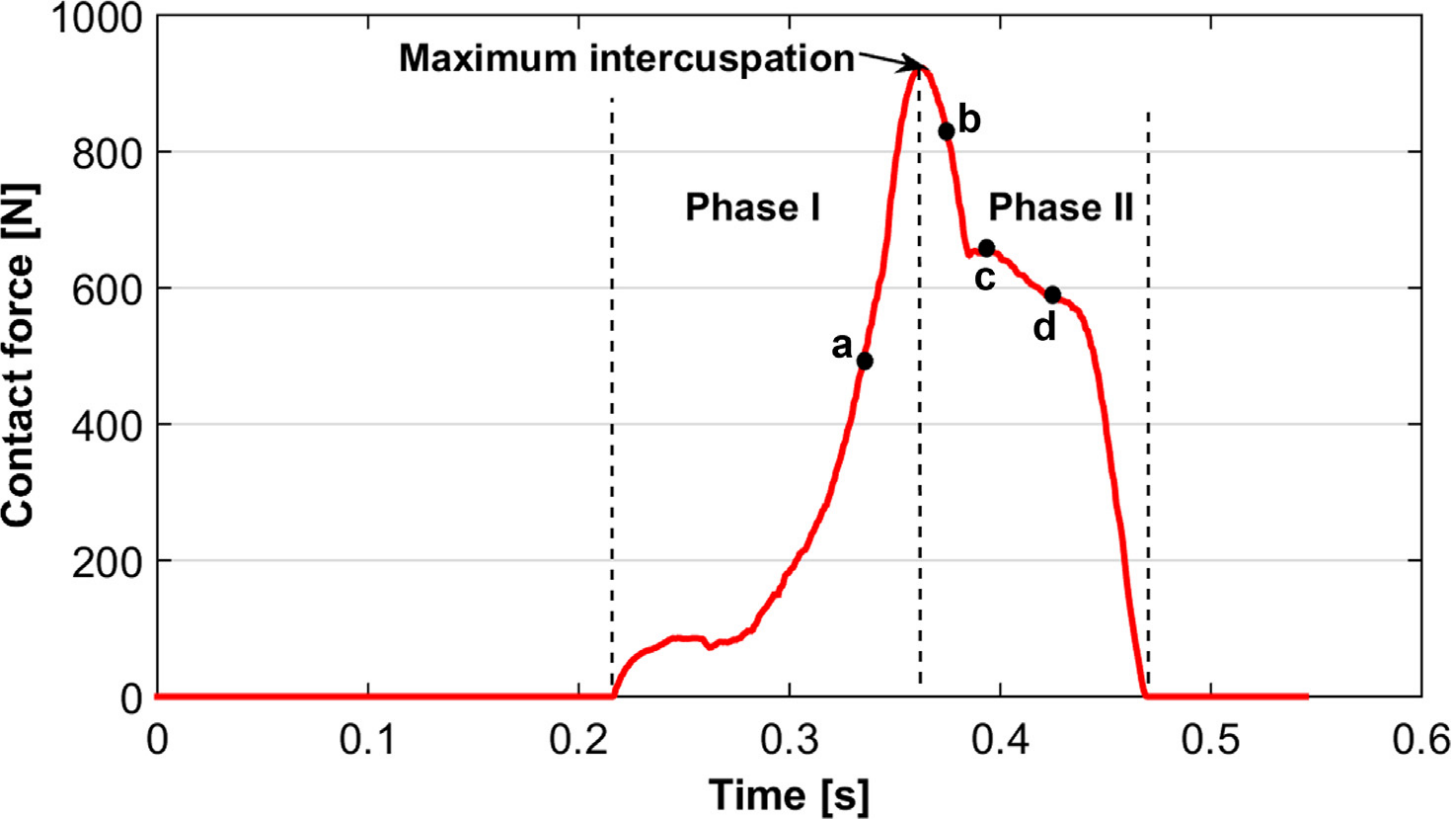
Contact forces (N) obtained during the non-linear dynamic FE crash colliding test. The four points in the plot correspond to the time steps at 0.335s (a), 0.375s (b), 0.394s (c) and 0.425s (d) discussed in the text and in Figs 8 and 9.

The maximum contact force achieved by solving the system is 923N (Fig 5). The force increased during phase I, reaching its greatest magnitude during maximum intercuspation contact. Even though the force reduced during phase II, values remained relatively high (Fig 5).

**Fig 6:**
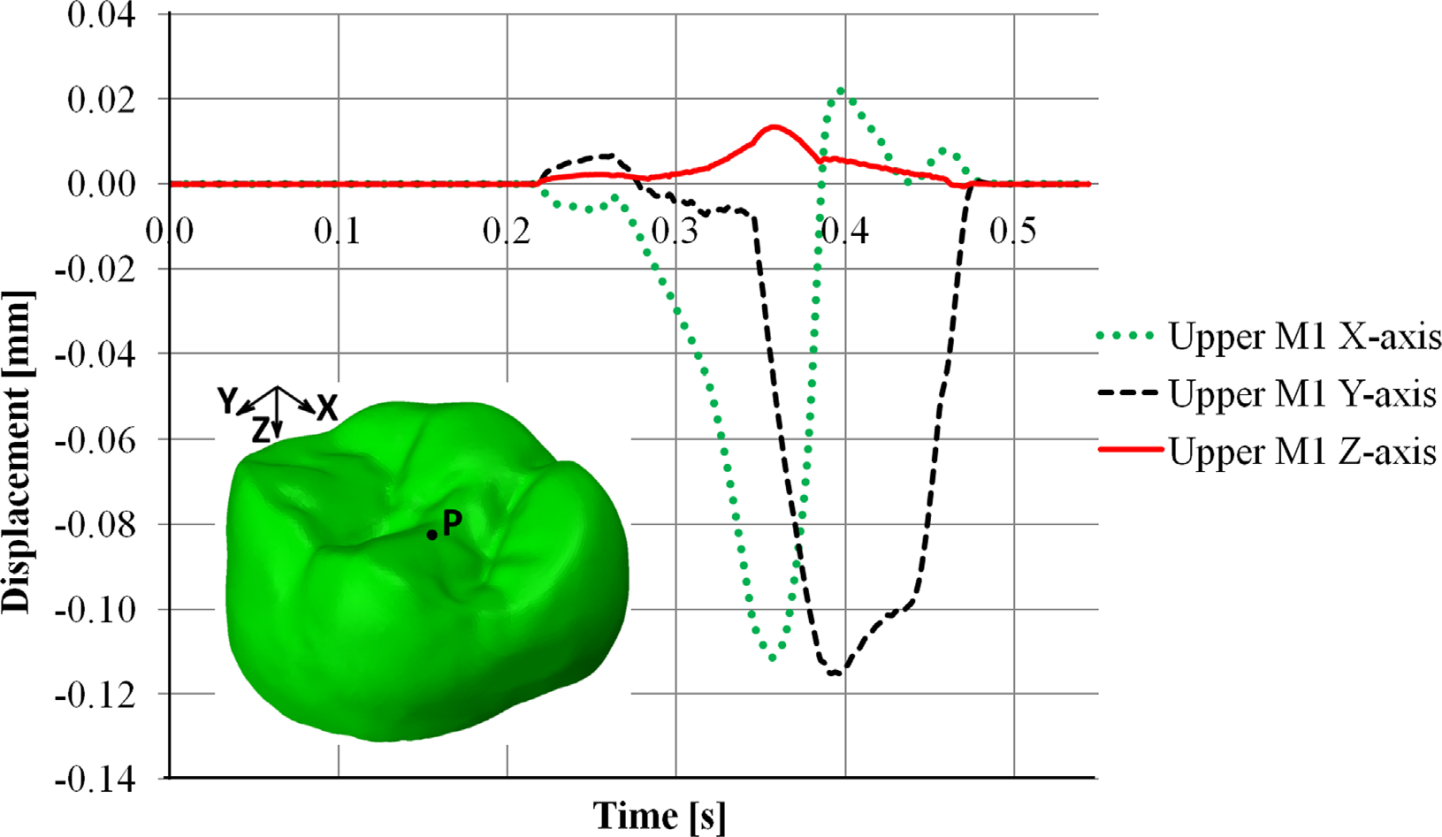
Displacement of the RM^1^. Displacement at a point (P), randomly selected in the occlusal surface of the RM^1^, during the non-linear dynamic FE crash colliding test. Note that the x-axis corresponds to the mesiodistal direction, the y-axis to the buccolingual direction and the z-axis to the crown-root direction.

**Fig 7:**
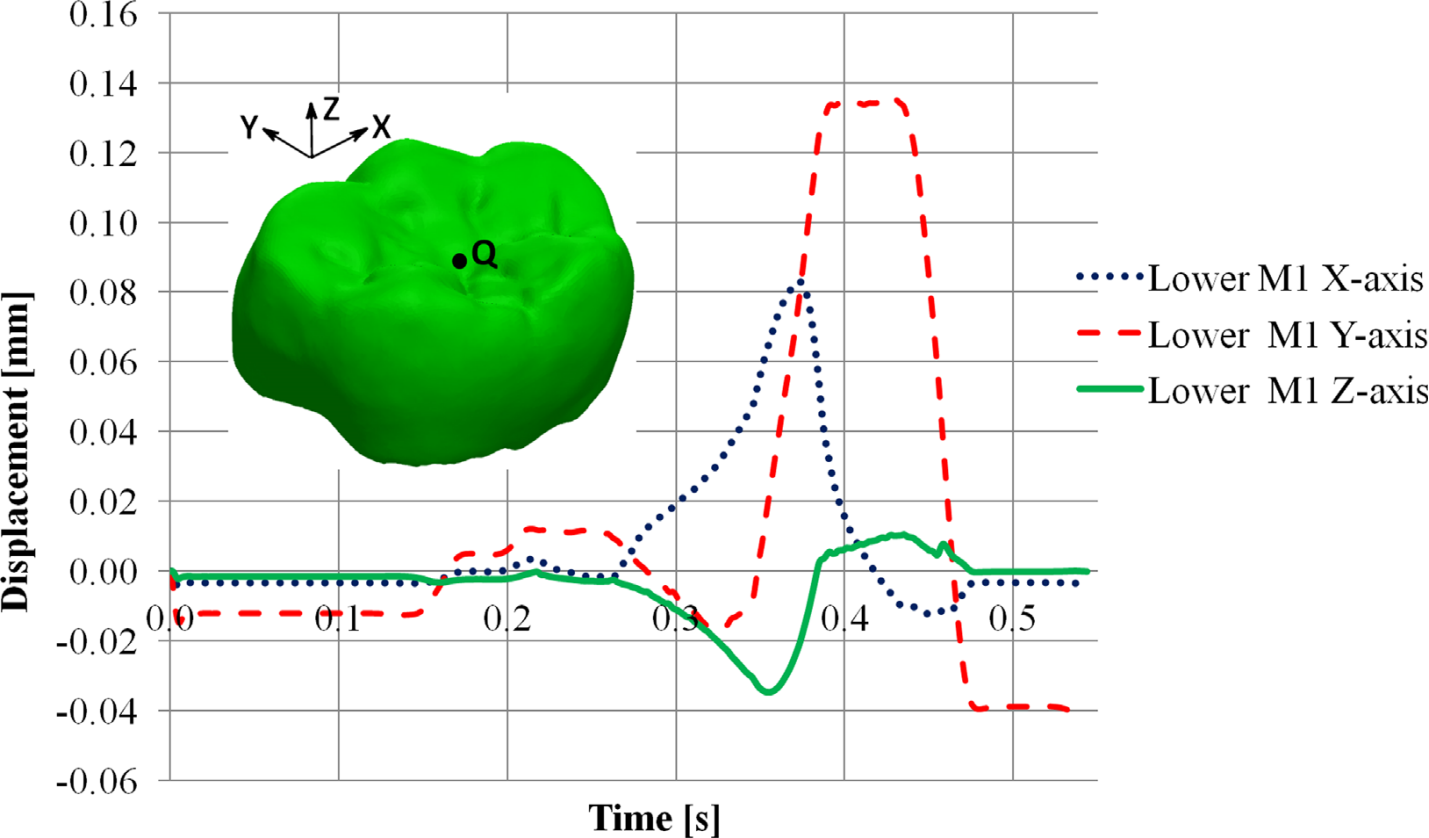
Displacement of the RM_1_. Displacement at a point (Q), randomly selected in the occlusal surface of the RM_1_, during the non-linear dynamic FE crash colliding test. Note that the x-axis corresponds to the mesiodistal direction, the y-axis to the buccolingual direction and the z-axis to the crown-root direction.

The displacements of the RM^1^ and RM_1_ along x-, y- and z-axes are summarized in Figs 6 and 7, respectively. For the RM^1^, the larger contact areas on the lingual cusps displaced the tooth mainly in the negative direction of the x- and y-axes, i.e. mesiolingually (Fig 6; S2 Video).

Due to dynamic effects the displacements of the RM_1_ during the closing and opening phases are different from zero. However, during the power stroke the dynamic effects were constrained by contacts between antagonists, and the RM_1_ displaced in the positive direction of the x- and mainly y-axes, i.e. distobuccally (Fig 7).

**Fig 8:**
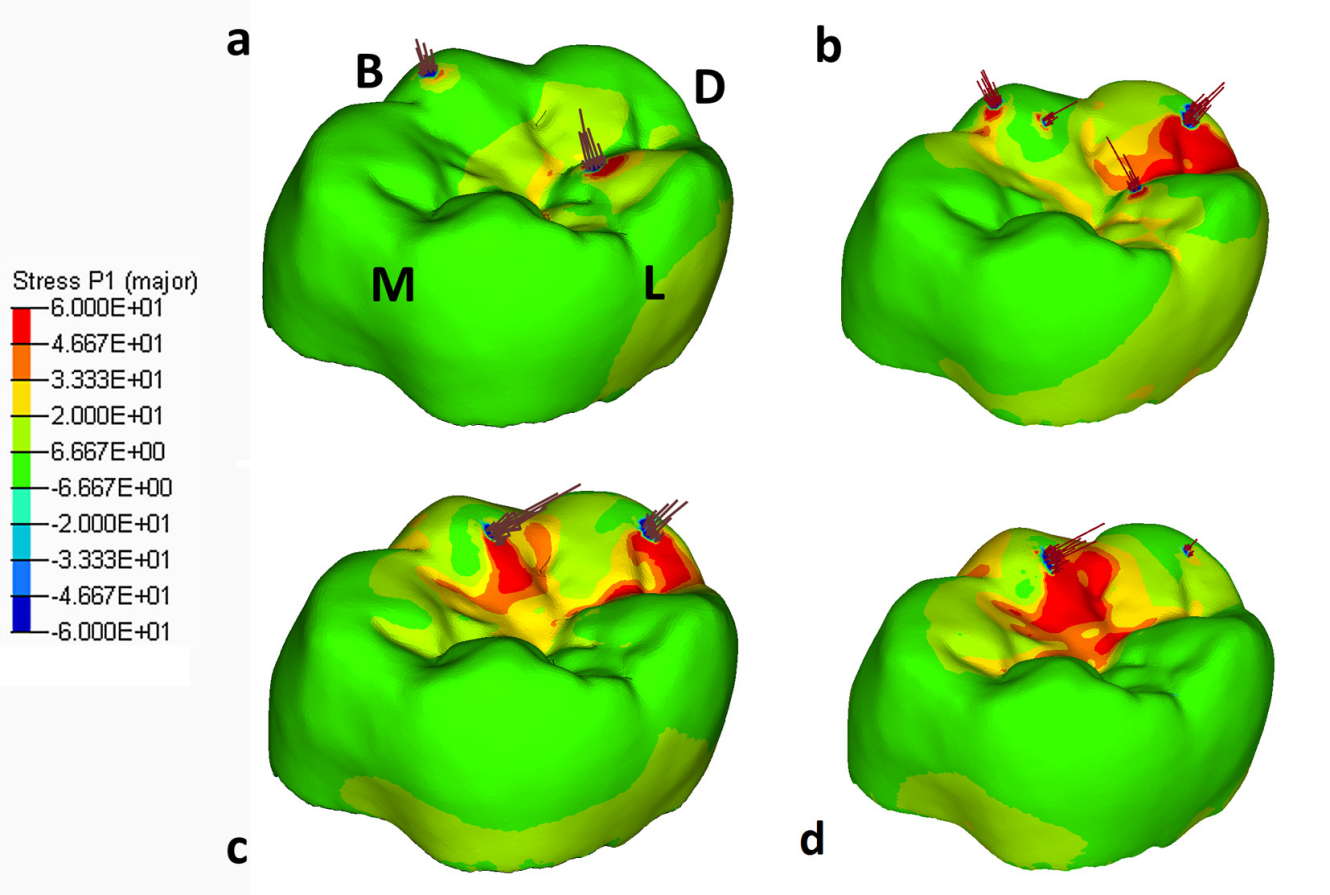
**The maximum principal stress distribution observed in RM**_**1**_ **at 0.335s (a), 0.375s (b), 0.394s (c) and 0.425s (d)**. See Fig 5 for the corresponding times during the power stroke. Arrows on the enamel are elemental contact forces. B = buccal; D = distal; L = lingual; M = mesial.

**Fig 9:**
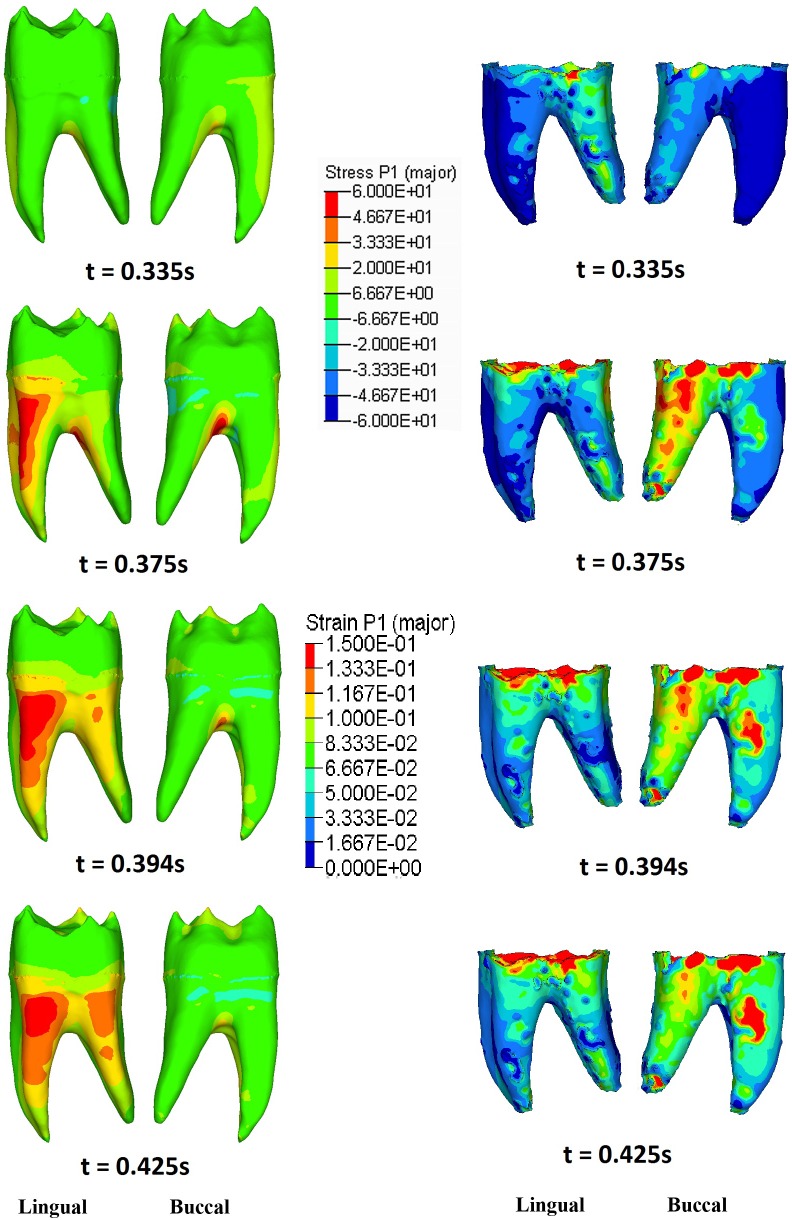
The maximum principal stress distribution observed in RM_1_ dentine (left) and the maximum principal strain distribution observed in RM_1_ PDL (right) at 0.335s, 0.375s, 0.394s and 0.425s. See Fig 5 for the corresponding times during the power stroke.

The distribution of maximum principal stresses on the enamel followed the same pattern for both RM_1_ and RM^1^. Contact pressure causes tensile stresses concentrated in the grooves of the occlusal surface, and their magnitude and area of interest reached its peak during maximum intercuspation (Fig 8; S3 Video). At mid-time of the power stroke (t ≈ 0.375s) the RM_1_ is mostly moving in the negative direction of the y-axis, causing high contact pressure and a sudden increase of tensile stresses on the distal cusps (mainly the hypoconulid), affecting also the grooves that surround these cusps (Fig 8B). As a consequence, tensile stresses increased also in the mesiolingual wall of the root (Fig 9) and, to a lower extent, in the superior alveolar margin of the mandible (S6 Fig; S3 Video). In the PDL tensile strains were highest in the distobuccal region (Fig 9).

Towards the end of the closing cycle, the RM_1_ continues moving in the negative direction of the y-axis, leading to maximum contact pressure on the hypoconid (at t ≈ 0.425s). Consequently, tensile stresses increased in the occlusal basin of the crown (Fig 8), in the lingual aspect of the root (Fig 9) and, more generally, in the upper half of the cortical bone of the mandible (S6 Fig). As far as the PDL is concerned, tensile strains remained higher in the buccal side than in the lingual one (Fig 9).

## Discussion and Conclusions

Currently, most of the FE simulations of teeth are limited to static loading conditions [7, 12, 13, 53], which reflect only a partial view of the dental hard and soft tissues’ functional response. Few studies combine FE methods and dynamic loading conditions [19, 20], but the analyses were restricted to coarse volumetric meshes that simplify the complex dental architecture and, importantly, did not envision the physiological sequential changes of occlusal contacts occurring during the power stroke. Yet, this information is pivotal to simulate realistic loading conditions.

The dynamic approach presented here is the logical advance following recent contributions that combined FE methods and occlusal macrowear analysis using high resolution digital models of teeth and dental supporting tissues [14, 15, 53, 54]. Using a non-linear dynamic FE crash colliding test based on the trajectory computed in the OFA software, we were able to compute tensile stresses and displacements of the FE models during the complete power stroke. The maximum contact force registered solving the system (923N) is in agreement with maximum values obtained during *in vivo* bite force measurements [55–58]. Moreover, our findings support previous FE studies that suggest both the importance of the PDL for load transfer to the alveolar wall [59] and the increase of tensile stresses in the upper half of the mandible, mainly in the lingual side [53].

This approach will be very useful in research pertaining to clinical dentistry, because the individual direction and position of occlusal forces are important factors for, e.g., estimating the long-term success of dental and endodontic treatments, dental implant designs, dental prostheses. A precise and individual occlusal surface reconstruction is crucial for a functional and balanced stress distribution in the dental arches with an optimum chewing efficiency, during the maintenance phase for patients with periodontitis, as well as for an understanding of the etiology of some very common dental pathologies, such as non-carious cervical lesions and abnormal tooth wear [60–62]. Moreover, our dynamic approach will enhance interpretations of investigative results related to evolutionary biology of teeth, e.g. to unravel the adaptive processes and significance of dental and resulting masticatory variations in respect to the development of various dental features, e.g. such as protostylid and mid-trigonid crests as well as enamel thickness and root shape.

It is important to note that the large size of the FE models (which is pivotal to maintain a detailed representation of the various tissues) and the complex computation to solve the non-linear equations require powerful computer facilities and is very time consuming. We are confident, however, that future technological advancements will address this practical issue, ultimately making the entire process less demanding and allowing us to implement food items between the occlusal surfaces in the simulation as well. Ultimately, experimentally derived *in vitro* or *in vivo* data on tooth function are required to substantiate our findings.

## Supporting Information

S1 Fig Energy ratio. The ratio between the total energy of the system and the initial total energy and the external energy.
(TIF)

S2 Fig Kinetic, Internal and Contact energy (convergence towards quasi-static equilibrium). (TIF)

S3 Fig Displacement comparison between coarse and fine mesh. Comparison of the displacement values between coarse mesh and fine mesh at a single point (P), randomly selected in the occlusal surface of the RM^1^, during the non-linear dynamic FE crash colliding test. Note that the x-axis corresponds to the mesiodistal direction, the y-axis to the buccolingual direction and the z-axis to the crown-root direction.
(TIF)

S4 Fig Comparison of internal energy computed using the “coarse” and the “fine” mesh. (TIF)

S5 Fig Contact force computed using the “coarse” and the “fine” mesh. (TIF)

S6 Fig The maximum principal stress distribution observed in the mandible at 0.335s, 0.375s, 0.394s and 0.425s. See Fig 5 for corresponding times during the power stroke.
(TIF)

S1 Text Supporting information. Verification of FE methodology.
(DOC)

S1 Video The power stroke. Simulation of the individual occlusal “power stroke” of the right mandibular and maxillary first molar applying the Occlusal Fingerprint Analyser (OFA) software.
(AVI)

S2 Video Displacement of the upper first molar. Displacement of the RM^1^ during the non-linear dynamic FE crash colliding test.
(AVI)

S3 Video Maximum principal stress distribution. The distribution of maximum principal stresses in the RM_1_ during the non-linear dynamic FE crash colliding test.
(AVI)
