# Supplementary figures and images for: Dynamic Modelling of Tooth Deformation Using Occlusal Kinematics and Finite Element Analysis
Source: PLoS One. 2016 Mar 31;11(3):e0152663. doi: 10.1371/journal.pone.0152663 (PMC4816422; doi:10.1371/journal.pone.0152663)

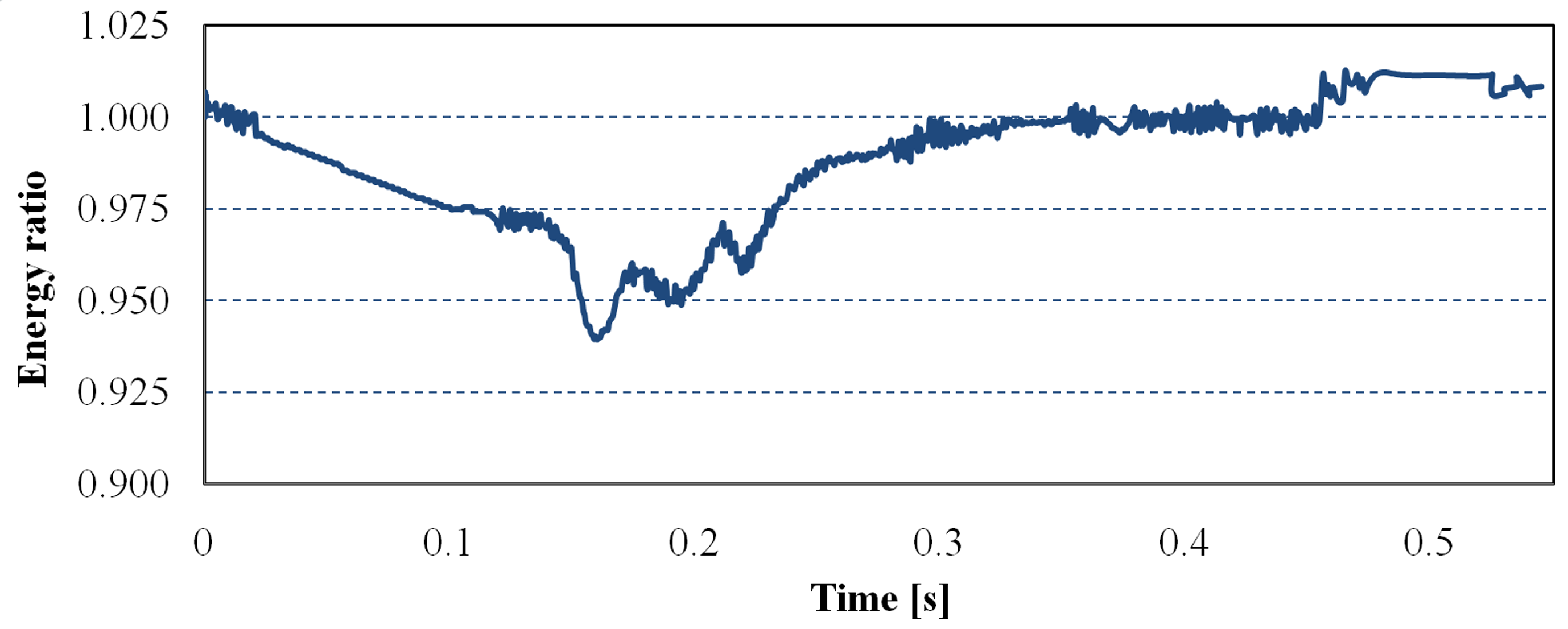

Supplement: S1 Fig — The ratio between the total energy of the system and the initial total energy and the external energy. (TIF) [file pone.0152663.s001.tif]

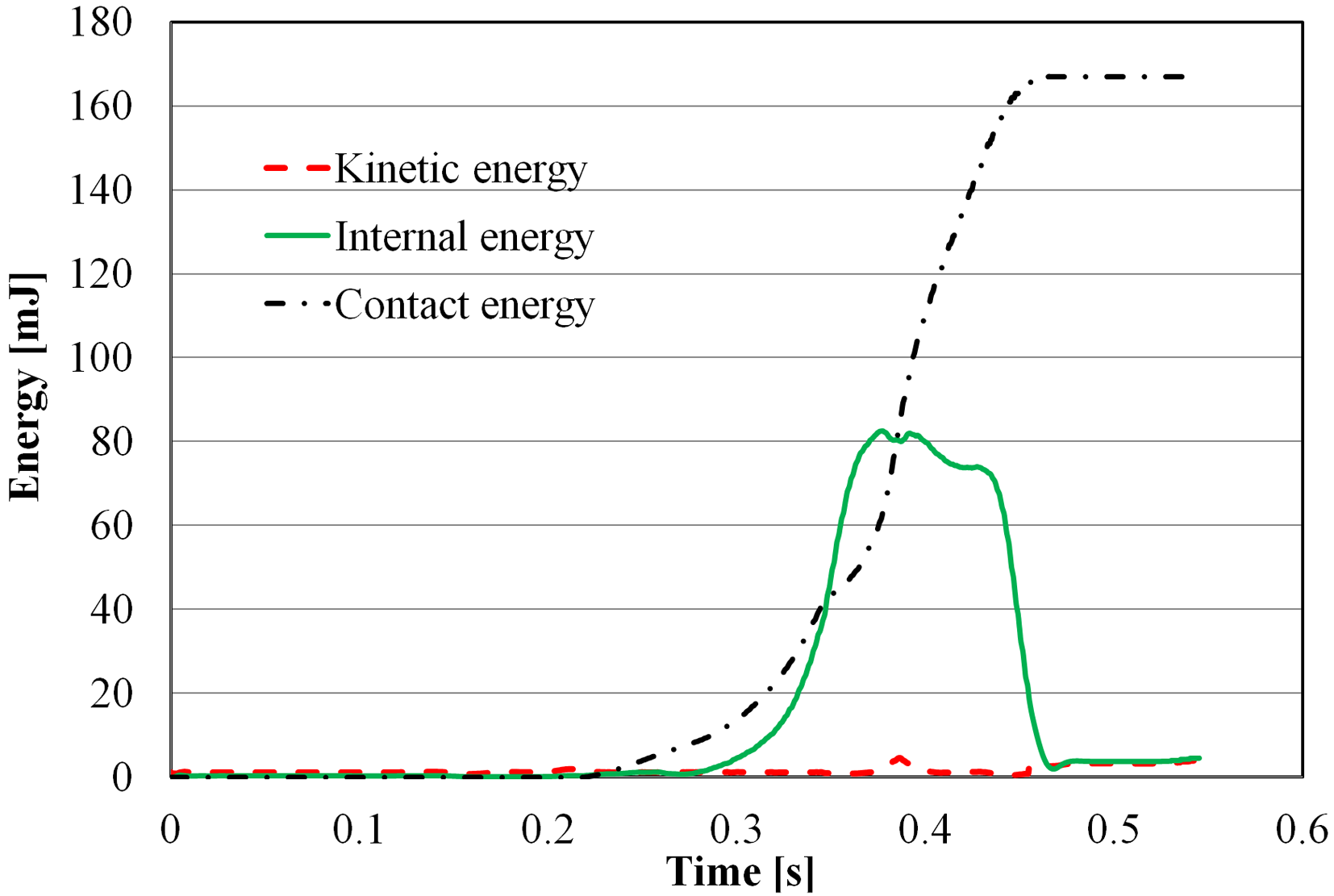

Supplement: S2 Fig — (TIF) [file pone.0152663.s002.tif]

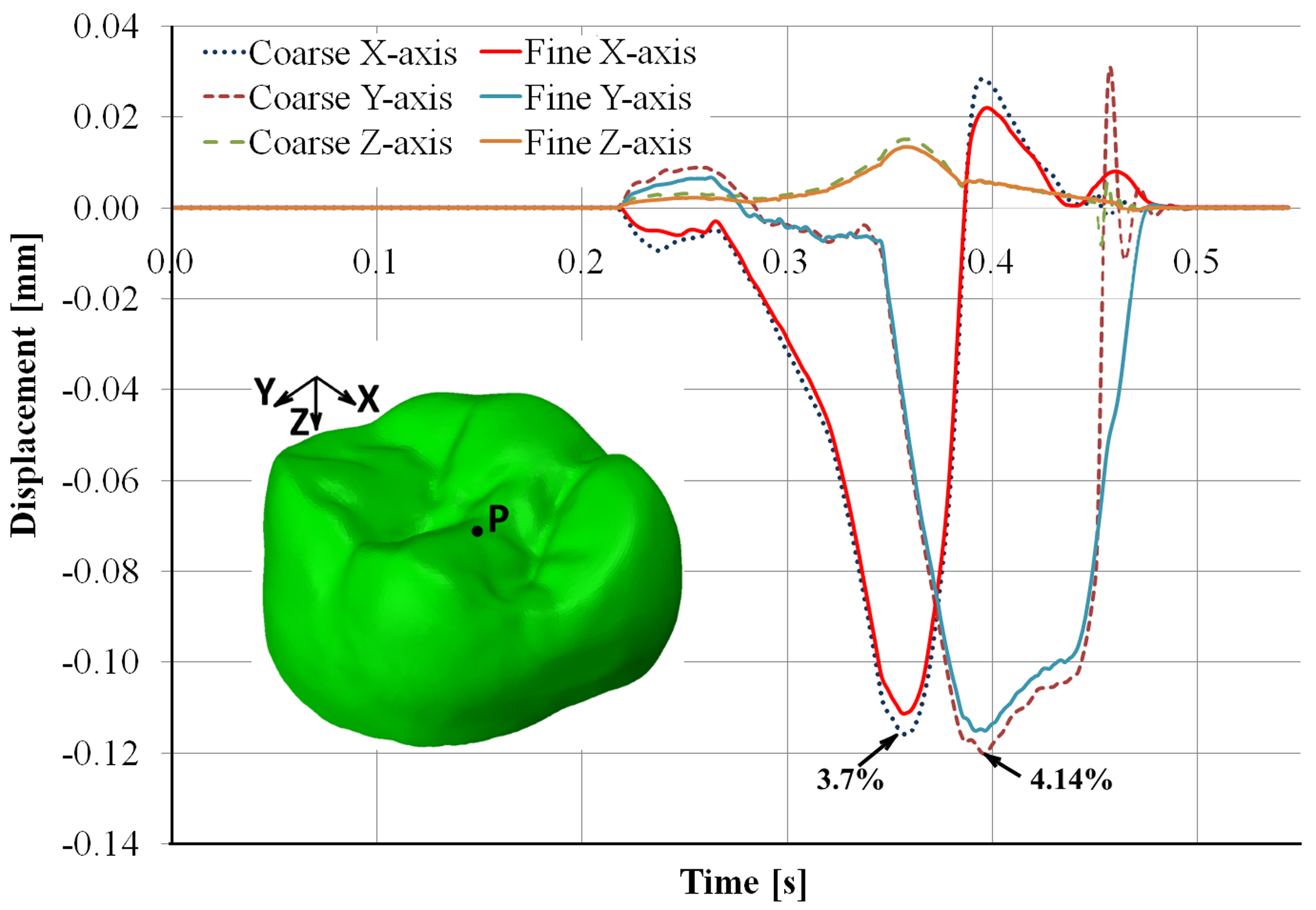

Supplement: S3 Fig — Comparison of the displacement values between coarse mesh and fine mesh at a single point (P), randomly selected in the occlusal surface of the RM1, during the non-linear dynamic FE crash colliding test. Note that the x-axis corresponds to the mesiodistal direction, the y-axis to the buccolingual direction and the z-axis to the crown-root direction. (TIF) [file pone.0152663.s003.tif]

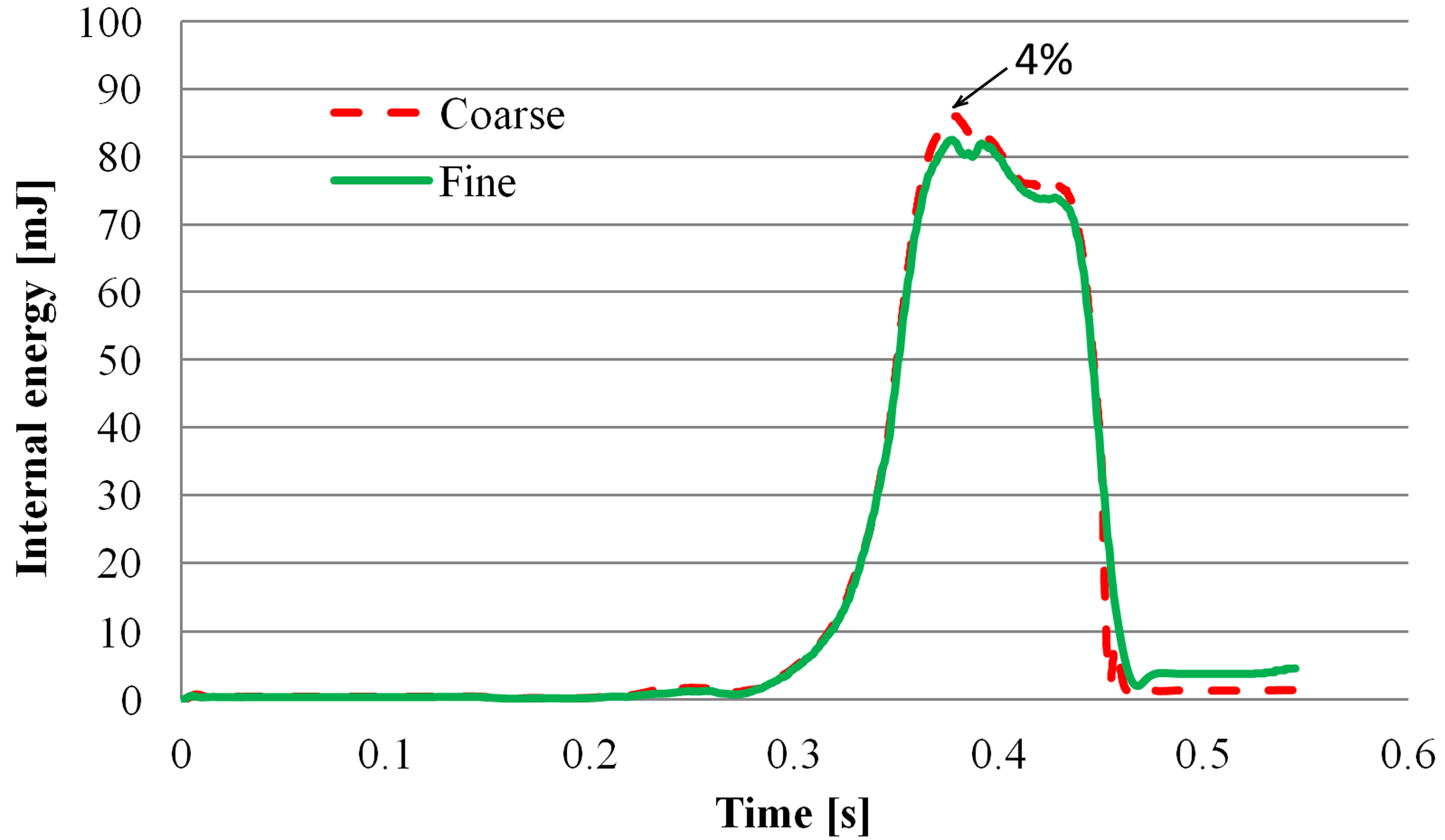

Supplement: S4 Fig — (TIF) [file pone.0152663.s004.tif]

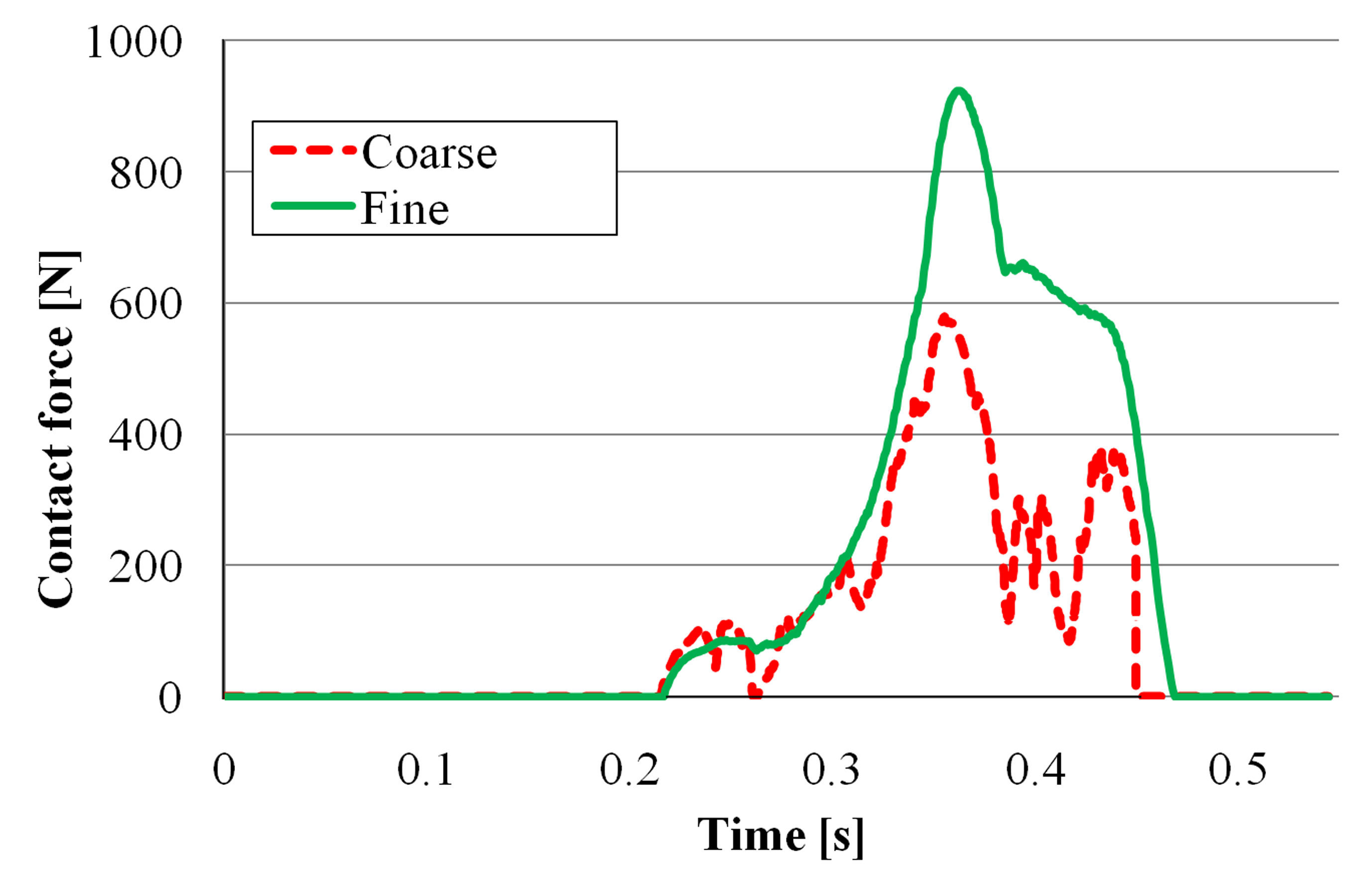

Supplement: S5 Fig — (TIF) [file pone.0152663.s005.tif]

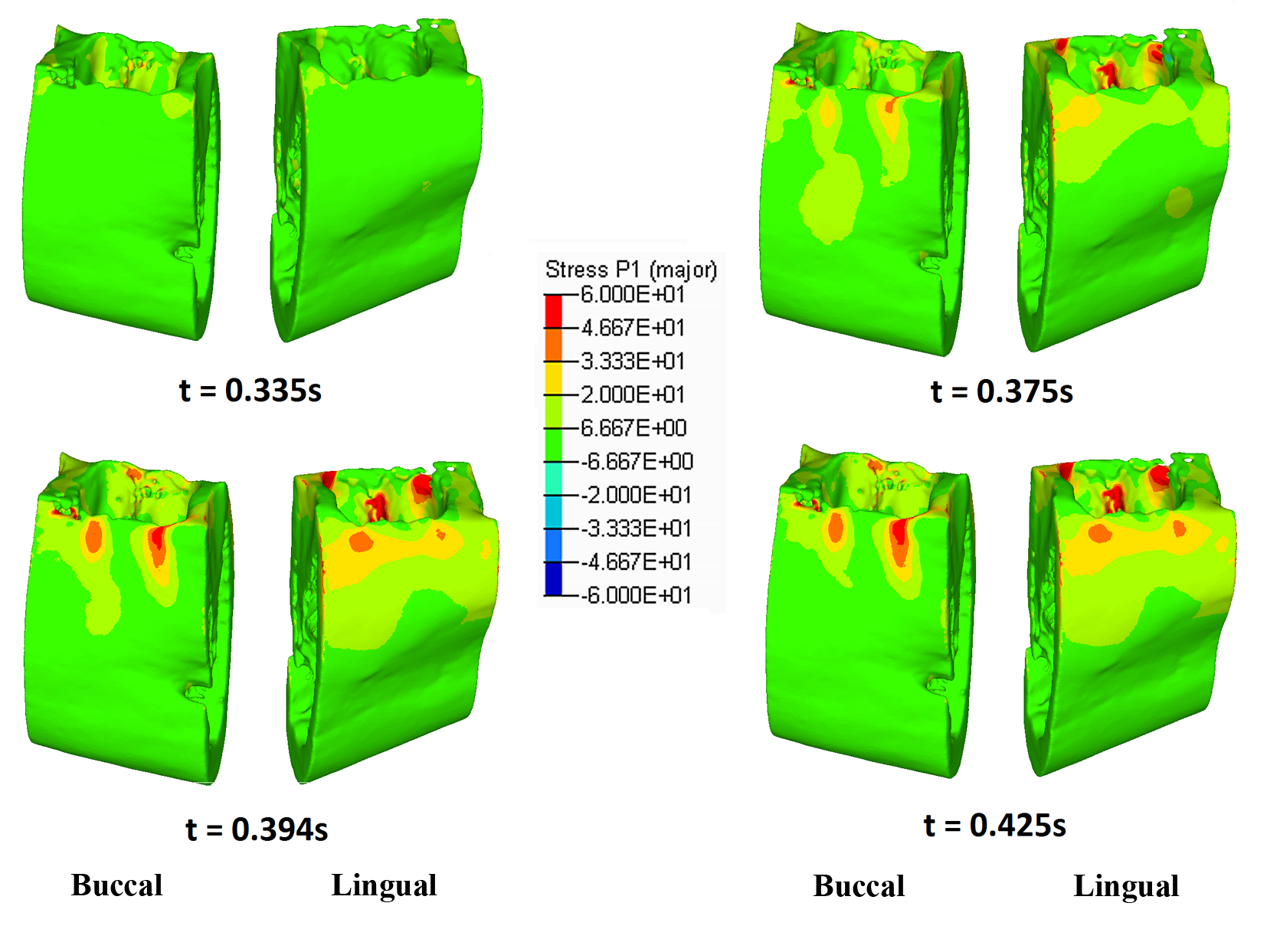

Supplement: S6 Fig — See Fig 5 for corresponding times during the power stroke. (TIF) [file pone.0152663.s006.tif]
