# Supplementary material for: Dynamic Modelling of Tooth Deformation Using Occlusal Kinematics and Finite Element Analysis
Source: PLoS One. 2016 Mar 31;11(3):e0152663. doi: 10.1371/journal.pone.0152663 (PMC4816422; doi:10.1371/journal.pone.0152663)
Supplement: S1 Text — Verification of FE methodology. (DOC) [file pone.0152663.s007.doc]

**Supporting Information**

**Verification of FE methodology**

**Energy balance**

The energy balance is tested through the expression:

*E_i_ + E_k_ + E_c_ + E_h_ = E_w_ + E_i0_ + E_k0_*

where: - *E_i_*: internal energy (IE); - *E_k_*: kinetic energy (KE); - *E_c_*: contact energy (CE); - *E_h_*: Hourglass energy (HE); - *E_w_*: External energy (EE); lower script “0” denotes the initial values.

The left hand side accounts for the total energy of the system, whereas the right hand side represents the initial total energy and the external energy. Energy ratio is the ratio of the left hand side to the right hand side. When the energy ratio is equal to unity, results of the simulation are considered fine. A ratio larger than unity reveals numerical instability or a failed contact. A ratio smaller than unity suggests that the energy was either artificially absorbed by hourglassing or ill-condition of contact. In our simulation, the minimum and maximum energy ratio ranges between 0.940 and 1.01 (S1 Fig**)**, confirming that there is not any abnormality in the analysis.

**Ratio KE/IE**

Both mass scaling and time scaling, which were used to accelerate the simulation, might lead to inertial effects. Hence, the scaling must assure that the kinetic energy is an order of magnitude lower than 5% of the internal energy (Hughes et al., 2013). Due to inertial effect caused by scaling, at the beginning of the closing phase the kinetic energy is not equal to zero (S2 Fig**)**. However, the average of kinetic energy during the power stroke (1.3 mJ) is around 5% of internal energy (1.4 mJ). This result ensures that the time scaling does not affect the accuracy of the simulation and that the power stroke is a quasi-static process.

**Time history of IE, KE and CE**

The change of the IE reflects loading rate. In this case loads are contact force and inertial forces. During the closing (non-contact) phase of the chewing cycle (t<0.15s), the IE is equal to zero. The IE should be different from zero during the power stroke (S2 Fig).

The KE is considered as an error when simulate a quasi-static problem by the explicit method. The KE reflects the loading sequence and the applied damping. At the beginning the KE slightly fluctuates due to inertial effects, but thanks to the applied damping (**C**), the KE is stable after a short time. The KE magnitude also changed when the contact force attained maximum value (at t~0.38s) or just after the power stroke, when the contact between enamels released (at t~0.45s). However these changes are of low entity and they do not affect the outcome of the simulation (S2 Fig).

The CE was positive, as expected in the case of a frictional contact. When compared with other energies, the contact energy was definitively important. The enamel to enamel contact represented the main cause of tooth deformation. Hence, the contact energy was equal to zero in the close phase and different from zero during the power stroke, similar to the IE (S2 Fig). The contact energy curve was smooth, confirming the contact was working normally. Even using a coarse mesh, which might lead to failed contacts, there was no abrupt change of CE. Deformation caused by gravity was small comparing to the deformation caused by contacts. Similarly, deformation caused by inertial effects was not important during the power stroke, because spurious oscillations were diminished when there are contacts between teeth.

**Convergence**

The convergence was verified by comparing displacement of the enamel and internal energy between “coarse” and “fine” meshes with respect to a point on the occlusal surface of the M^1^ enamel (the M^1^ was selected because not affected by scaling techniques). In general, differences in displacements were negligible (S3 Fig), except when t > 0.45s due to differences in enamel mesh size. With the “fine” enamel mesh, the process of M_1_ enamel coming in/out of contact with the antagonist enamel surface was smooth (i.e., a smooth mechanical behavior for the “fine” mesh model). With the “coarse” enamel mesh, the changes of contact areas are less smooth, leading to sudden changes of contact force.

The maximum difference of internal energy between “coarse” and “fine” mesh simulations was low, about 4% (S4 Fig), confirming the convergence of the results. It is important to note, however, that using the “coarse” mesh the contact force curve is discontinuous at some points (i.e., at a specific time-step contact the resultant contact force is the sum of all elemental contact forces, which indeed in the “coarse” mesh are not smooth) (S5 Fig).
